# Trends in assisted ventilation and outcome for obstructive pulmonary disease exacerbations. A nationwide study

**DOI:** 10.1371/journal.pone.0171713

**Published:** 2017-02-03

**Authors:** Anne Pernille Toft-Petersen, Christian Torp-Pedersen, Ulla Møller Weinreich, Bodil Steen Rasmussen

**Affiliations:** 1 Department of Clinical Medicine, Aalborg University, Aalborg, Denmark; 2 Department of Health, Science and Technology, Aalborg University, Aalborg, Denmark; 3 Department of Respiratory Diseases, Aalborg University Hospital, Aalborg, Denmark; 4 Department of Anaesthesia and Intensive Care, Aalborg University Hospital, Aalborg, Denmark; National Yang-Ming University, TAIWAN

## Abstract

**Background:**

Non-invasive ventilation (NIV) has been used for decades in treatment of exacerbations of chronic obstructive pulmonary disease (COPD). The impact of the changing use of assisted ventilation in acute exacerbations on outcomes has not been fully elucidated and we aimed to describe these changes in the Danish population and describe their consequences for mortality.

**Methods:**

A register-based study was conducted of a cohort of 12,847 patients admitted for acute exacerbation of COPD (AECOPD) from 2004 through 2011, treated with invasive mechanical ventilation (IMV) or NIV for the first time. Age, sex, in-hospital mortality rates, time to death or readmission for AECOPD were established and changes over time tracked.

**Results:**

The number of admissions for AECOPD where assisted ventilation was used was 1,130 in 2004 and had increased by 145% in 2011. First time ventilations increased by 88%. This was mainly due to an increase in use of NIV accounting for 36% of the total number of assisted ventilations in 2004 and 67% in 2011. The number of IMV with or without NIV treatments remained constant. The mean age of NIV patients increased from 71.5 to 73.6 years, but remained constant at 70.0 years in IMV patients. Mortality rates both in hospital and after discharge for patients receiving NIV remained constant throughout the period. In-hospital mortality following IMV increased from 30% to 38%, but mortality after discharge remained stable.

**Conclusion:**

Assisted ventilation has been increasingly used in a broader spectrum of AECOPD patients since the introduction of NIV. The changes in treatment strategies have been followed by shifts in in-hospital mortality rates following IMV.

## Introduction

Positive pressure ventilation without endotracheal intubation has been deployed for a number of different conditions for most of a century[1]. Within the last three decades especially bi-level positive airway pressure has been used in treatment of hypercapnic respiratory failure due to chronic obstructive pulmonary disease (COPD). Concomitantly, the annual number of treatments with assisted ventilation given in hospitals has increased [2,3].

The use of non-invasive ventilation (NIV)/bi-level positive airway pressure ventilation, in patients with acute exacerbations of COPD (AECOPD) and hypercapnic respiratory failure has been recommended in clinical guidelines worldwide [4]. Guidelines are based on several randomised trials of NIV vs. no assisted ventilation in this disease entity; a meta-analysis from 2003 estimated a rather consistent in-hospital mortality reduction from 22% with the standard medical therapy to 9% with NIV [5]. To our knowledge, only 2 randomised trials have compared mortality following NIV and invasive mechanical ventilation (IMV) in severe AECOPD, and none of these trials demonstrated a significant mortality reduction [6,7]. In propensity-matched analyses, NIV has been shown to compare favourably to IMV but comorbidity and pneumonia at admission reduce the association [8,9].

Observational studies of the effect of NIV in respiratory medical units and in intensive care units (ICUs) have shown higher mortality rates compared to estimates inferred from randomised trials of NIV vs standard medical treatment. Among these observational studies, a British audit carried out in 2008 revealed an in-hospital mortality of 25% among AECOPD patients treated with NIV. In addition, a subgroup analysis demonstrated, that patients who would have been eligible for the largest of the trials included in the meta-analysis [5] had an in-hospital mortality rate of 26% [10,11]. Among Danish patients with AECOPD treated with NIV, 30 days mortality has been stagnant at 24–25% from 2005 through 2011[12,13]. Lately, an Italian study conducted in 2013 had higher mortality rates than the randomised trials, with an in-hospital mortality of 18% [14].

Several factors of importance for mortality in relation to AECOPD have been established, among those age, burden of comorbidity, level of consciousness at admission, nutritional status, severity of AECOPD, severity of stable phase lung function impairment, previous AECOPDs, presence of pneumonia and–in case of initial NIV—escalation to IMV [10,15–20]. Importantly, some of these characteristics were exclusion criteria in the trials leading to NIV endorsement.

The use of NIV and IMV in daily clinical practise has not been fully described. Likewise, it remains uncertain whether patients undergoing NIV and IMV for the first time in later years have prognoses different from the prognoses of those enrolled just after endorsement of NIV as a ventilatory mode of choice.

Based on the existing literature, we hypothesized that the introduction of in expansion in the use of NIV had 1) reduced the use of IMV for AECOPD, 2) increased the use of ventilation among patients of high age, and 3) been followed by an increase in mortality in connection with NIV. This study therefore had three aims: Firstly, to investigate hospitalized patients with AECOPD with regards to the overall tendency in the number of first time NIV and IMV treatments in Denmark. Secondly, to investigate changes in the age of the patients and, thirdly, to describe the in-hospital mortality, mortality after discharge from hospital and COPD related readmissions among first time NIV and IMV patients.

## Methods

All hospitalisations in the period from 1/1 2000 to 31/12 2012 were identified in the Danish National Patient Registry.

*AECOPD admissions* were defined as admissions with either a primary diagnosis of COPD (ICD-10: DJ44) or with a complex of either acute respiratory failure (ICD-10: DJ96) or pneumonia (ICD-10: DJ13-18) with COPD as a secondary diagnosis. ICD-10 diagnostic codes were used in Denmark throughout the study period. Admissions where the patients were less than 30 years old were excluded from the cohort to minimize inclusion of misclassified asthma [21]. The register does not allow establishing the temporal sequence of treatments, and the IMV+NIV group did thus encompass both NIV succeeded by IMV and IMV succeeded by NIV. Registrations of Nordic Medico-Statistical Committee (NOMESKO)[22] codes BGDA1 (non-invasive ventilation, in which continuous positive airway pressure is not included) and BGDA0 (invasive mechanical ventilation) registered at any point in time during the admissions were retrieved. The AECOPD admissions were labelled “IMV only”, “NIV only”, “IMV+NIV” or “no ventilation”, if only BGDA0, only BGDA1, both of these, or none of these, respectively, were registered. A compound category “IMV±NIV” included both “IMV” and “IMV+NIV”. *All AECOPD ventilations* were all AECOPD admissions where assisted ventilation was registered. *First time AECOPD admissions* were the first AECOPD admissions registered for any patient and *first time AECOPD ventilations* the first AECOPD admissions where assisted ventilation of any kind was performed in hospital. To assess the burden of comorbidity, Charlson Comorbidity Scores based on diagnoses made during hospitalizations and out-patient visits 10 or 5 years prior to the end of the hospitalization were calculated. Points were assigned to individual diseases ad modum Radovanovic [23] and grouped in 3 with 1, 2 or 3+ points respectively as per definition all patients received one point for having COPD. Dates of birth and death were obtained from the Danish Civil Registration System. For all AECOPD admissions, the age at admittance and whether the patient survived to discharge from index hospitalisation were registered. For patients discharged alive, follow up for evaluation of time to death started at discharge and continued until death or censure at 31/12 2012. No information on domiciliary assisted ventilation was obtained, as it was not used for COPD in Denmark in the time period of interest. *Readmissions* were subsequent AECOPD admissions with or without ventilation. Where readmission and death coincided, the patient was considered readmitted. Follow up for evaluation of time to readmission started at discharge from index hospitalisation and continued until the next readmission, death or censure at 31/12 2012. As NIV could not be registered in the National Patient Registry until 2004, we only examined AECOPD admissions from that year onward. Informed consent was not required according to Danish legislation.

Statistical analyses were performed with the SAS 9.4 software package (SAS institute Inc, Cary, NC, USA) and R software 3.2.2. Annual numbers of treatments were evaluated with Poisson regression and trends in average ages were examined by ANOVA. Chi-square tests were used for comparisons of mortality rates across treatment groups. Developments in in-hospital mortality rates (numbers of deaths/numbers of treatments) and mortality/readmission rates over time were examined with Poisson regression and presented as relative rates per 5 years. Models were adjusted for age (quintiles), sex and Charlson scores calculated over 10 years (0, 1, 2, >2). In a sensitivity analysis, we used Charlson scores calculated over 5 years. Linearity of annual changes was visually assessed by comparing the linear estimate with the trend of values for each year. P-values below 0.05 were considered significant.

Retrospective use of register data does not require ethical approval in Denmark. The Danish Data Agency gave permission to use the data (GEH-2014-018, I-Suite nr: 02736).

## Results

**Fig 1:**
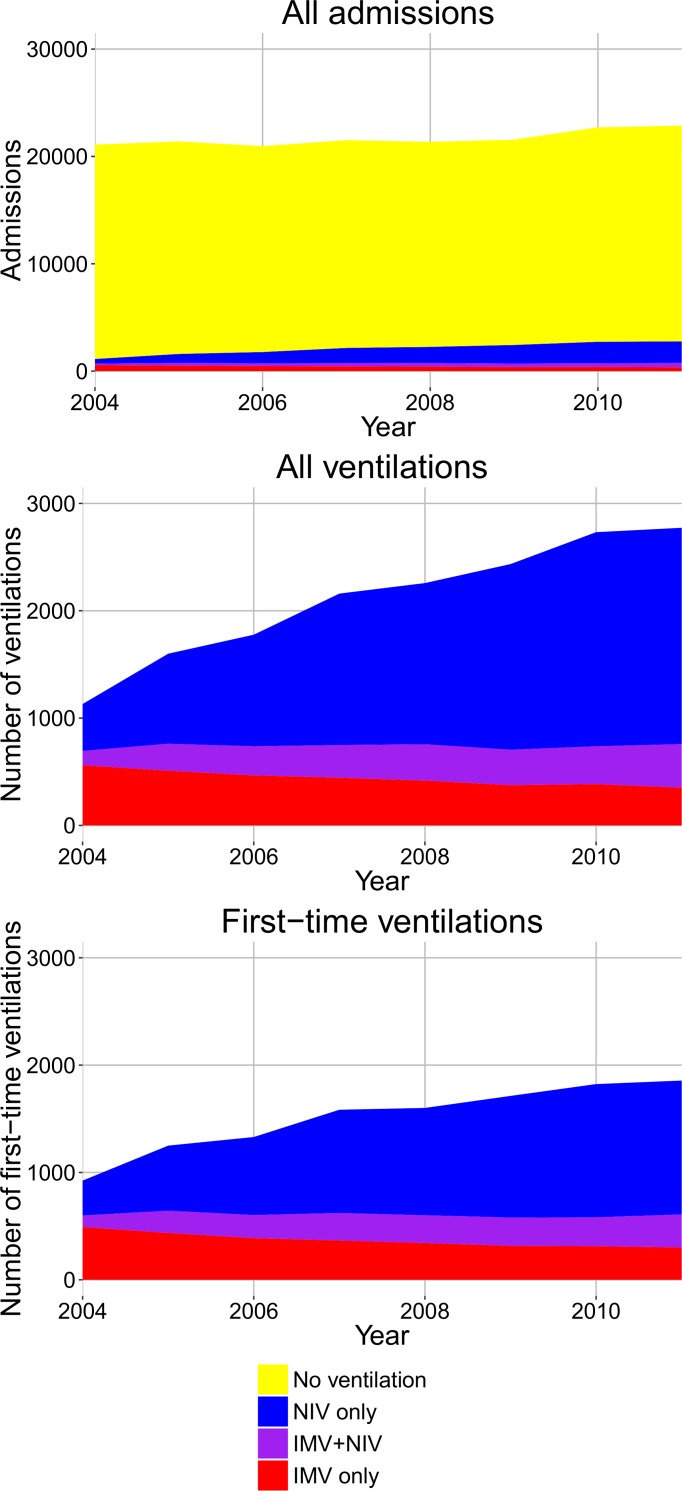
The annual number of AECOPD admissions. A: All AECOPD admissions. B: AECOPD admissions were ventilation was performed. C: AECOPD admissions where the patient had not been ventilated for AECOPD before.

From 2004 through 2011, there were 173,456 admissions to Danish hospitals for AECOPD, ventilation was used in 9.7% of these admissions. The male-to-female-ratio was 45/55 and the median age was 73.7 (IQR 66–80) years. The annual numbers of admissions to Danish hospitals due to AECOPD from 2004 to 2011 are displayed by mode of ventilation in Fig 1, panel A. The total annual number of admissions increased 8.3%, and the proportion of admissions where no ventilation was performed decreased from 95% to 88%. Fig 1, panel B shows only the admissions where ventilation was performed. The annual number of treatments with NIV only increased, 4.6-fold from 2004 to 2011 and, likewise, the annual number of treatments with combined NIV and IMV increased, 3.1-fold. The annual number of treatments with IMV as the only mode of assisted ventilation decreased by 40% from 2004 to 2011, but the total number of IMV treatments increased slightly by 9.4%. Fig 1, panel C shows only first-time COPD admissions with ventilation. The use of NIV only increased 3.8-fold and the combined use of NIV and IMV increased 2.9-fold. The use of IMV only decreased by 40% but, again, the total use of IMV remained comparatively stable (increased 2%). Patients who had both NIV and IMV made up a decreasing proportion of NIV patients (25% in 2004 and 20% in 2011).

**Fig 2:**
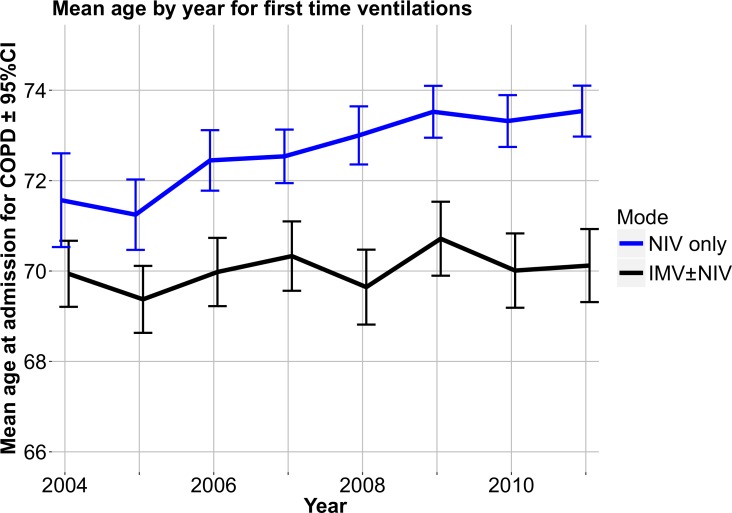
Development in the mean ages of patients treated with assisted ventilation by mode of ventilation.

The mean age of first-time ventilated patients is depicted in Fig 2. The mean age of NIV only patients increased by 2.1 (0.8–3.2, p = 0.001) years from 2004 to 2011 (p for trend <0.001), but no significant change was detected in mean ages for patients receiving IMV with or without NIV (p = 0.24).

Case mix is displayed in Table 1 and average mortality rates in Table 2. Among first-time ventilated patients, in-hospital mortality rates were higher among patients receiving IMV in any combination than among patients who only received NIV (p<0.001). In contrast, mortality rates after discharge were higher among patients receiving only NIV (p<0.001).

**Table 1:** Baseline characteristics of first time ventilated patients by mode of ventilation.

	First time ventilated	
	NIV alone	IMV±NIV
N	7,242	4,839
Age, years (sd)	72.9 (9.9)	70.0 (9.8)
Male, N (%)	4,281 (59.1)	2,528 (52.2)
**Charlson comorbidity index**		
1, N (%)	2,964 (40.9)	1,775 (36.7)
2, N (%)	1,813 (25.0)	1,165 (24.1)
3+, N (%)	2,465 (34.0)	1,899 (39.2)

**Table 2:** Mortality rates with 95% confidence intervals in the cohort of patients admitted for AECOPD.

	All admitted	All ventilated	First time ventilated	
			NIV alone	IMV±NIV
**Admitted**				
Died in-hospital, %	6.8 (6.7–6.8)	24.2 (24.1–24.3)	24.4 (24.3–24.5)	33.7 (33.6–33.8)
Died within a year of admission, %	32.7 (32.6–32.7)	52.8 (52.7–52.8)	50.9 (50.8–51.0)	50.9 (50.8–51.1)
**Discharged alive**				
Died within a month, %	4.4 (4.4–4.4)	6.3 (6.3–6.4)	6.1 (6.0–6.1)	3.3 (3.3–3.4)
Died within a year, %	25.9 (25.9–25.9)	28.5 (28.5–28.6)	26.4 (26.3–26.5)	17.2 (17.1–17.3)

Table 3 shows the development in in-hospital mortality rates among first-time ventilated patients. In univariate analyses, mortality among NIV only patients did not change significantly, whereas in-hospital mortality among patients receiving IMV with or without NIV increased over time (from 32.2% in 2004 to 37.6% in 2011). The increase remained after adjustment for age, sex and Charlson score…

**Table 3:** Rate ratio per 5 years calendar time of in-hospital mortality by mode of ventilation.

	NIV only	IMV±NIV
Odds ratios for death per 5 years		
Unadjusted	1.03 (0.92–1.15)	1.16 (1.04–1.28)
Adjusted†	0.96 (0.85–1.07)	1.12 (1.01–1.25)

†Adjusted for age, sex, and Charlson score

In univariate analyses of mortality and mortality and/or readmission after discharge as well as in analyses adjusted for age, sex and Charlson score, no changes over time were discovered (Table 4).

**Table 4:** Rate ratio per 5 years calendar time of death or readmission within a year mortality by mode of ventilation.

	NIV only	IMV±NIV
Odds ratios for death within a year per 5 years		
Unadjusted	1.06 (0.95–1.18)	1.02 (0.88–1.18)
Adjusted†	1.00 (0.90–1.12)	0.99 (0.85–1.15)
Odds ratio for death/readmission within a year per 5 years		
Unadjusted	1.02 (0.92–1.13)	1.08 (0.97–1.21)
Adjusted†	1.04 (0.93–1.16)	1.09 (0.98–1.22)

†Adjusted for age, sex, and Charlson score

Exchanging Charlson scores calculated over 10 years for scores calculated over 5 years did not affect the outcomes either in hospital or after discharge (S1 and S2 Tables).

## Discussion

The present study shows that the annual numbers of assisted ventilations and first-time ventilations have increased since 2004, driven by an increase in the number of NIV only treatments and treatments where IMV and NIV was combined. The total number of IMV treatments has been relatively stable. In the years following introduction of NIV, the average first-time ventilated recipient became older, but in-hospital mortality remained stable throughout the study period, as did mortality after discharge. In-hospital mortality among patients receiving IMV treatment increased whereas mortality after discharge was constant.

Like other observational studies, we found a far higher mortality rate with NIV than seen in the randomised trials. That NIV has been used in patients who would have been excluded from the trials is a likely explanation but the finding suggests that a large and increasing number of patients are treated “off-label” and to some extent without evidence of improved outcomes or outcomes comparable to IMV. Future studies are needed to establish when NIV and IMV respectively might benefit elderly patients with multiple comorbidities. The increased use of NIV seen in our study seems to have taken place wherever the technique has been introduced [3,15,17,24]. Nonetheless, the constant use of IMV in our population contrasts to the decrease found in most other studies.

Our approach differ from that of most other studies [3,15,17] in that we evaluated the overall use of different modes of assisted ventilation and not only the use in patients never ventilated before. This might explain our finding of an almost unaltered use of IMV. A Danish study covering the last three years of our study period did, in line with our findings, not detect any decrease in the use of IMV [13]. The study period was, however, short and did not include the years where most of the increase in the number of NIV treatments took place. That the recommendation of NIV has had no impact on the use of IMV in Denmark in contrast to other countries might have several explanations. NIV is being delivered in respiratory medical units as well as in ICUs and Danish rates of assisted ventilation for admitted COPD patients are comparatively low [13], which might reflect either high admission rates or restrictive use of assisted ventilation compared to other countries. A Spanish study, in which the proportion of admitted patients who received ventilation was smaller than what we found and the predominance of NIV over IMV much larger, detected a decreasing use of IMV [24]. However, different registration makes international comparison difficult.

It might be that NIV in Denmark does not decrease the need of IMV because patients treated with NIV are mainly selected among patients whose acute condition does not warrant IMV or whose age and frailty contraindicated assisted ventilation. Lending support to this is the increasing age of NIV only patients. Alternatively, the availability of ICU beds, a prerequisite for initiation of IMV, may have influenced the chosen mode of ventilation. The extent to which ICU capacity influences decisions to admit patients to ICU is controversial as is the consequences of such influence [25–27]. The ICU capacity in Denmark has been rather stable. It is possible that more beds in ICUs have been allocated to patients with poor prognoses over time, because the capacity for NIV treatment of other patients have been enlarged in respiratory, intermediary and medical wards. Inherently observational, our data does only allow conjectures of causality.

In our study, in-hospital mortality among patients receiving NIV only remained stable throughout the study period whereas mortality among patients receiving IMV treatment increased. Large, register based studies from USA [15] and Spain [28], respectively, found decreasing mortality rates among patients receiving NIV only. In concordance with our method, assisted ventilation administered in ICU as well as wards was considered. However, the American study had a lower baseline mortality rate, 9%, and none of the studies limited their study population to first time ventilated patients making comparison of mortality over time difficult as the proportion of repeatedly ventilated patients increases.

Less afflicted patients having been transferred to the NIV group and patients with a poorer prognosis a priori being left in the IMV group might explain the increasing in-hospital mortality among IMV patients in our study. This would implicate that other patients whose prognosis would previously have limited the level of care are introduced in the IMV group, as the number of treatments is stable. This argument could be supported by recent studies suggesting that clinicians’ opinions on the benefit conferred by active treatment have become more optimistic in later years. As such, the SAPS II score of COPD patients admitted to French ICUs from 1998 through 2010 increased more than 4 points [29] and in Austria, ICU admission due to COPD increased by about two thirds from 1998 and 2008 [30].

Mortality and readmission rates after discharge did not significantly change over the study period. This suggests that, although the population of patients receiving ventilation has increased and might over time have included patients with increasingly severe acute illness, terminal COPD is still considered a contraindication to ventilation. A Danish study of patients where the registered cause of death was COPD showed that only 24% were treated with NIV in their last 6 months [31]. Our data suggests that the increasing number of ventilations after the introduction of NIV has not been associated with an increase in the proportion of ventilated patients whose death cannot be deferred. Opposite trends where a general improvement in community care for COPD patients balances an increasing disease burden might also explain the stable rates.

There are noteworthy strengths and limitations to our study. Among the strengths is the reliability of the Danish medical registers which is well established for a number of diagnoses [32], among those the COPD diagnosis applied here (which had a positive predictive value of 92%) [21], procedures [33] and vital status [34]. Unique person identifiers virtually eliminate loss to follow-up from reasons other than emigration. In addition, unique person identifiers allow first-time ventilated patients to be considered separately, which arguably diminishes bias introduced by changing case mixes. Another strength of our study is our inclusion of treatments irrespective of where they were given. Over time, NIV has been introduced in some respiratory and general wards. We include alike in our study ICU and ward-based NIV treatments. The present GOLD guidelines provide recommendations regarding admission to either ICU or ward and regarding choice of ventilator mode[4], but the extent to which these guidelines are complied with is unknown, likely variable and likely to depend on predefined advancement decisions. Although distinction between ward and ICU based ventilation would have been useful for comparisons with other studies of the outcome of NIV, we suspect that changes in the pattern of ICU referral would introduce bias.

The most noteworthy limitation in our study is the lack of information on the case mix. As in most large-scale observational studies, we do not have access to data on the stable phase lung function impairment prior to the AECOPD and are thus unable to determine whether the case mix with regards to COPD stage has shifted over time. Likewise we cannot establish the presence of advancement orders, which are common in Denmark in this particular patient group [35]. Arguably, the proportion of NIV patients with limited level of care is likely to increase along with an increase in the availability of NIV. The generalisability of our results to other countries where prevalence, case mix, health care organisation and attitudes to end-of-life might be very different cannot directly be ascertained. Another limitation is our assumption that the number of first-time ventilations reflects the probability of ventilation for any given COPD patient. The premises for this assumption are a constant prevalence of COPD and an unchanging case mix of the COPD population in the years 2003 to 2011. This is, however, an approximation. Previous Danish studies have found that the incidence of hospitalisations decreased in spite of a constant prevalence of COPD that had at some point required hospitalisation in the period from 2002–2009 [36], the incidence of hospitalisations decreased. Along with this, the use of intensive care, the burden of comorbidity, all-cause previous hospital admissions and mortality increased, thereby prompting suggestions that the severity of disease among patients hospitalised for COPD were increasing [37].

## Conclusion

The present study shows that, in Denmark, the increased use of NIV for exacerbations of COPD has not been accompanied by a decrease in the use of IMV. NIV treatment has been initiated in increasingly older patients without changes in either in-hospital or after discharge mortality. In-hospital mortality among patients treated with IMV has increased but mortality after discharge has remained stable.

## Supporting information

S1 Table Rate ratio per 5 years calendar time of in-hospital mortality by mode of ventilation. Charlson comorbidity index was calculated based on hospital contacts within the last 5 years.
(DOCX)

S2 Table Rate ratio per 5 years calendar time of death or readmission within a year mortality by mode of ventilation. Charlson comorbidity index was calculated based on hospital contacts within the last 5 years.
(DOCX)
