# Supplementary material for: Trends in assisted ventilation and outcome for obstructive pulmonary disease exacerbations. A nationwide study
Source: PLoS One. 2017 Feb 3;12(2):e0171713. doi: 10.1371/journal.pone.0171713 (PMC5291443; doi:10.1371/journal.pone.0171713)
Supplement: S1 Table — Charlson comorbidity index was calculated based on hospital contacts within the last 5 years. (DOCX) [file pone.0171713.s001.docx]

S1 Table.

|  | **NIV only** | **IMV±NIV** |
| --- | --- | --- |
| Odds ratios for death per 5 years |  |  |
| Unadjusted | 1.03 ( 0.92 - 1.15 ) | 1.16 ( 1.04 - 1.28 ) |
| Adjusted† | 0.96 ( 0.86 - 1.08 ) | 1.12 ( 1.00 - 1.24 ) |

† Adjusted for age, sex, and Charlson score
