# Supplementary material for: Trends in assisted ventilation and outcome for obstructive pulmonary disease exacerbations. A nationwide study
Source: PLoS One. 2017 Feb 3;12(2):e0171713. doi: 10.1371/journal.pone.0171713 (PMC5291443; doi:10.1371/journal.pone.0171713)
Supplement: S2 Table — Charlson comorbidity index was calculated based on hospital contacts within the last 5 years. (DOCX) [file pone.0171713.s002.docx]

S2 Table.

|  | **NIV only** | **IMV±NIV** |  |
| --- | --- | --- | --- |
| Odds ratios for death within a year per 5 years | |  |  |
| Unadjusted | 1.06 ( 0.95 - 1.18 ) | 1.02 ( 0.88 - 1.18 ) |  |
| Adjusted† | 1.01 ( 0.91 - 1.12 ) | 0.99 ( 0.85 - 1.15 ) |  |
| Odds ratio for death/readmission within a year per 5 years | | |  |
| Unadjusted | 1.02 ( 0.92 - 1.13 ) | 1.08 ( 0.97 - 1.21 ) |  |
| Adjusted† | 1.03 ( 0.93 - 1.15 ) | 1.09 ( 0.98 - 1.21 ) |  |
|  | | | |

† Adjusted for age, sex, and Charlson score
